# Mean airway pressure as an independent risk factor for developing acute kidney injury in mechanically ventilated critically ill patients: a multi-center retrospective analysis

**DOI:** 10.1080/0886022X.2026.2675108

**Published:** 2026-05-24

**Authors:** Ying Gao, Jiayan Zhang, Xiaomeng Xue, Yanchao Su, Qinghe Yan, Fuhong Su, Yipeng Fang, Keliang Xie

**Affiliations:** a Department of Critical Care Medicine, Tianjin Medical University General Hospital, Tianjin, China; b Experimental Laboratory of the Department of Intensive Care, Erasme Hospital, Université Libre de Bruxelles, Brussels, Belgium; c Department of Anesthesiology, Tianjin Institute of Anesthesiology, Tianjin Medical University General Hospital, Tianjin, China

**Keywords:** Mean airway pressure, acute kidney injury, mechanical ventilation, critically ill patients, retrospective study, multi-center analysis

## Abstract

Mechanical ventilation is a well-recognized risk factor for acute kidney injury (AKI), but the specific role of mean airway pressure (P_mean_) remains insufficiently defined. We conducted a multicenter retrospective cohort study including 17,428 mechanically ventilated patients from the MIMIC-IV and eICU-CRD databases. To evaluate the association between P_mean_ within the first 24 h of ventilation and subsequent AKI, we applied machine learning–based feature selection, multivariable regression, propensity score matching (PSM), and subgroup analyses. Across all analytical approaches, P_mean_ consistently emerged as the most influential ventilatory parameter associated with AKI. A non-linear dose–response relationship was observed, with threshold effects at approximately 8.8 cmH_2_O in the MIMIC-IV cohort and 10.0 cmH_2_O in the eICU-CRD cohort. Beyond these inflection points, each 1-cmH_2_O increase in P_mean_ was associated with significantly higher risks of AKI and adverse outcomes, including mortality and the need for renal replacement therapy. These associations remained robust across regression models, PSM, and multiple subgroup analyses. Collectively, our findings indicate that elevated P_mean_ is independently associated with AKI risk in critically ill patients receiving mechanical ventilation. The identification of clinically relevant inflection points provides a pragmatic exposure definition that may inform risk stratification and the design of future prospective and randomized studies evaluating kidney-conscious ventilatory strategies.

## 1. Introduction

Acute kidney injury (AKI) represents a major clinical challenge in critical care medicine, with reported incidence rates of 5–7.5% among hospitalized patients and up to 20% in intensive care unit (ICU) populations [1–3]. AKI development is independently associated with substantially worse clinical outcomes, including a 50–60% increase in ICU mortality, accelerated progression of chronic kidney disease, and prolonged ICU length of stay, resulting in significant healthcare resource utilization [4,5].

Mechanical ventilation is a well-established risk factor for AKI in critically ill patients [6,7], primarily through hemodynamic compromises induced by elevated airway pressures [8]. However, the relative importance of specific ventilatory parameters remains unclear. P_mean_ is determined by peak inspiratory pressure, positive end-expiratory pressure (PEEP), and the inspiratory-to-expiratory time ratio, which are dynamic and real-time characteristics and represents the average alveolar pressure throughout the respiratory cycle [9]. Increased P_mean_ appears to influence cardiac output [10], CVP [11], and intra-abdominal pressure [12], potentially creating a cascade of hemodynamic alterations that could predispose to AKI development.

In our analysis, machine learning–based feature selection identified P_mean_ as the most influential ventilatory parameter associated with adverse outcomes, prompting us to specifically examine its relationship with AKI. To investigate this potential association between P_mean_ and AKI, we conducted a comprehensive multicenter retrospective cohort study utilizing two large, independent critical care databases (MIMIC-IV and eICU-CRD) to systematically evaluate P_mean_ as an independent risk factor for AKI in mechanically ventilated patients.

## 2. Material and methods

### 2.1. Data sources and study design

This multi-center retrospective cohort study utilized data from two large, publicly available critical care databases: the Medical Information Mart for Intensive Care IV (MIMIC-IV, version 2.2) and the eICU Collaborative Research Database (eICU-CRD). MIMIC-IV contains de-identified clinical data from over 70,000 ICU admissions at Beth Israel Deaconess Medical Center from 2008–2019, while eICU-CRD comprises records from 335 U.S. hospitals during 2014 to 2015. The study followed the Strengthening the Reporting of Observational Studies in Epidemiology (STROBE) guidelines for retrospective analyses. Both databases provide comprehensive ICU parameters, including ventilator settings, demographic parameters, comorbidities, laboratory results, and clinical outcomes. Ethical approval was obtained from the Institutional Review Boards (IRB) of Beth Israel Deaconess Medical Center (BIDMC) and the Massachusetts Institute of Technology (MIT), with a waiver of informed consent due to the de-identified nature of the data. Data access was granted by MIT and PhysioNet after completing the required National Institutes of Health (NIH) online training (Certification No. 43025968 for Yipeng Fang).

### 2.2. Population selection

We included adult patients (≥18 years) who received invasive mechanical ventilation in ICUs. The exclusion criteria were: (1) repeated ICU admissions (only the first ICU stay was analyzed); (2) no mechanical ventilation; (3) AKI diagnosis prior to or within 24 h of mechanical ventilation initiation; (4) missing essential ventilator.

### 2.3. Exposure and endpoint

The primary exposure was P_mean_, defined as the integrated average pressure applied throughout the respiratory cycle during mechanical ventilation. Ventilator parameters were obtained following the methodology of Yan Y et al. [13]. Specifically, P_mean_ was not derived from calculation but was directly extracted as recorded values from the clinical data streams. In the MIMIC-IV database, P_mean_ was sourced from the chart events table (item ID: 224697). In the eICU-CRD database, it was extracted from the respiratory charting table where resp-chart-value-label was recorded as ‘Mean Airway Pressure’. We extracted multiple P_mean_ value from the initial 24-h ventilation period following intubation, including the first recorded value, mean, maximum, and minimum P_mean_ levels, to comprehensively assess their associations with clinical outcomes. For analytical purposes, P_mean_ was evaluated both as a continuous variable (per 1-cmH_2_O increase) and categorically by quartiles (Q1 ≤ 7.8, Q2 7.8–8.7, Q3 8.7–10.3, Q4 > 10.3 cmH_2_O) based on interquartile ranges to examine potential threshold effects and dose-response relationships. All exposure variables were systematically extracted from the databases to ensure data consistency and minimize measurement bias.

The primary outcome was incident AKI occurring >24 h after mechanical ventilation initiation. AKI was diagnosed and staged according to the ‘Kidney Disease: Improving Global Outcomes’ (KDIGO) criteria, using serum creatinine changes only: (1) an increase in serum creatinine by ≥0.3 mg/dL (≥26.5 μmol/L) within 48 h, or (2) an increase to ≥1.5 times baseline within 7 days. Baseline creatinine was defined as the lowest value within 7 days before ICU admission or the first measurement at ICU admission if pre-admission data were unavailable. Secondary outcomes included: (1) AKI severity (stages 1–3); (2) renal replacement therapy requirement; (3) mortality outcomes (ICU, in-hospital, 28-day, and 90-day); and (4) ICU and hospital length of stay (LOS).

### 2.4. Data extraction

We collected comprehensive baseline data across multiple domains, including demographic characteristics, comorbidities, laboratory parameters, blood pressure, fluid input-output balance, specific interventions received, disease severity scores, and types of ICUs.

Demographic information included age, sex, and body weight. Complications were identified based on ICD codes at discharge and included coronary heart disease, heart failure, hypertension, diabetes mellitus, chronic pulmonary disease, liver disease, chronic kidney disease, anemia, malignant cancer, and kidney transplant.

Laboratory parameters were recorded as extreme values observed within the first 24 h following initiation of mechanical ventilation. These consisted of white blood cell (WBC) count, hemoglobin, platelet count, mean corpuscular volume (MCV), red cell distribution width (RDW), sodium (Na^+^), potassium (K^+^), anion gap (AG), oxygenation index (P/F ratio), partial pressure of carbon dioxide (PaCO_2_), lactate, glucose, blood urea nitrogen (BUN), and creatinine. Fluid balance was assessed based on total intake and output documented during the initial 24-h period post-ventilation.

We also extracted data on specific interventions administered, such as vasoactive agents, nephrotoxic medications, and diuretics. Nephrotoxic drugs were defined as those administered from hospital admission until the onset of AKI. This group included nephrotoxic antibiotics, nonsteroidal anti-inflammatory drugs (NSAIDs), dehydrating agents, contrast media, and immunosuppressants. Nephrotoxic antibiotics consisted of gentamicin, amikacin, tobramycin, vancomycin, sulfadiazine, and amphotericin. Contrast media referred to commonly used iodine-based and gadolinium-based agents. Immunosuppressants considered were cyclosporine and tacrolimus.

Disease severity was assessed using the maximum Sequential Organ Failure Assessment (SOFA) score, as well as the first recorded Acute Physiology Score III (APS III) and Acute Physiology and Chronic Health Evaluation II (APACHE II) scores.

### 2.5. Data cleaning

A rigorous data cleaning protocol was implemented to ensure analytical validity. Outlier detection was performed using Tukey’s method (1.5 × IQR threshold), with extreme values cross-referenced against clinical documentation – implausible values without clinical correlation were treated as missing. For variables with <10% missingness, median and mean imputation was applied to preserve data distribution. Variables with 10–20% missing values were imputed using random forest algorithms through missForest package in R, which accounts for complex variable interactions. Parameters exceeding 20% missingness were excluded from analysis to maintain reliability. Importantly, all ventilation-related variables underwent rigorous outlier detection, but no imputation was performed for all of them to prevent artificial introduction of bias in our primary exposure. Patients with missing ventilator data during the first 24 h after mechanical ventilation were excluded from final analysis.

### 2.6. Statistical analysis

Continuous variables were presented as medians with interquartile ranges (IQR) or means with standard deviations (SD) based on distribution normality assessed by Shapiro-Wilk tests, while categorical variables were reported as frequencies (percentages). For normally distributed variables, between-group comparisons were performed using Student’s t-tests (two groups) or ANOVA (multiple groups), while non-normally distributed variables were analyzed using Mann-Whitney U tests (two groups) or Kruskal-Wallis tests (multiple groups). Categorical variables were compared using χ^2^ tests or Fisher’s exact tests, as appropriate.

For feature selection, we employed three complementary algorithms: (1) Boruta algorithm, a wrapper method based on random forest, for all-relevant feature selection, (2) recursive feature elimination (RFE) with random forest predictive importance ranking according to logistic regression, and (3) LASSO regression with 10-fold cross-validation *via* L1-penalized coefficient score. The intersection of features identified by above three methods was visualized through Venn diagram, with P_mean_ emerging as one of the most robust biomarkers across modalities.

Nonlinear relationships between P_mean_ and the risk of AKI were examined using restricted cubic splines (RCS) with 4 knots placed at 0.05, 0.35, 0.65, and 0.95, testing for nonlinearity *via* likelihood ratio tests comparing models with linear and spline terms. Critical thresholds were identified where the 95% CI of odds ratios excluded 1.0.

Multivariable logistic regression models were constructed in three models: Model 1 adjusted for demographic factors (age, sex, weight); Model 2 additionally adjusted for comorbidities; Model 3 further incorporated interventions, disease severity scores, PaO_2_/FiO_2_ Ratio (P/F ratio) and PaCO_2_. Results were reported as adjusted odds ratios (aOR) with 95% confidence intervals.

To address potential confounding, propensity score matching (PSM) was performed using a 1:1 nearest-neighbor matching algorithm without replacement, with a caliper width of 0.05 standard deviations of the logit propensity score. The propensity score was estimated using logistic regression incorporating clinically relevant covariates, including demographic factors, comorbidities, incorporated interventions, disease severity scores, P/F ratio and PaCO_2_.

Subgroup analyses were conducted according to demographic factors, comorbidities, disease severity scores, P/F ratio and PaCO_2_. The type of ICU departments (medical ICU/cardiac care unit [MICU/CCU], mixed medical-surgical ICU [Med-SICU], and surgical ICU [SICU]) was included to partially account for potential confounding by surgical interventions. Interaction effects were assessed by incorporating multiplicative interaction terms between P_mean_ and each subgroup variable in the regression models, with statistical significance determined through likelihood ratio tests comparing models with and without the interaction terms.

All analyses were performed using R version 4.3.2 and Stata 13.0, with two-tailed *P*-values <0.05 considered statistically significant.

## 3. Results

### 3.1. Baseline information and clinical results from the MIMIC IV and eICU-CRD databases

**Figure 1.**
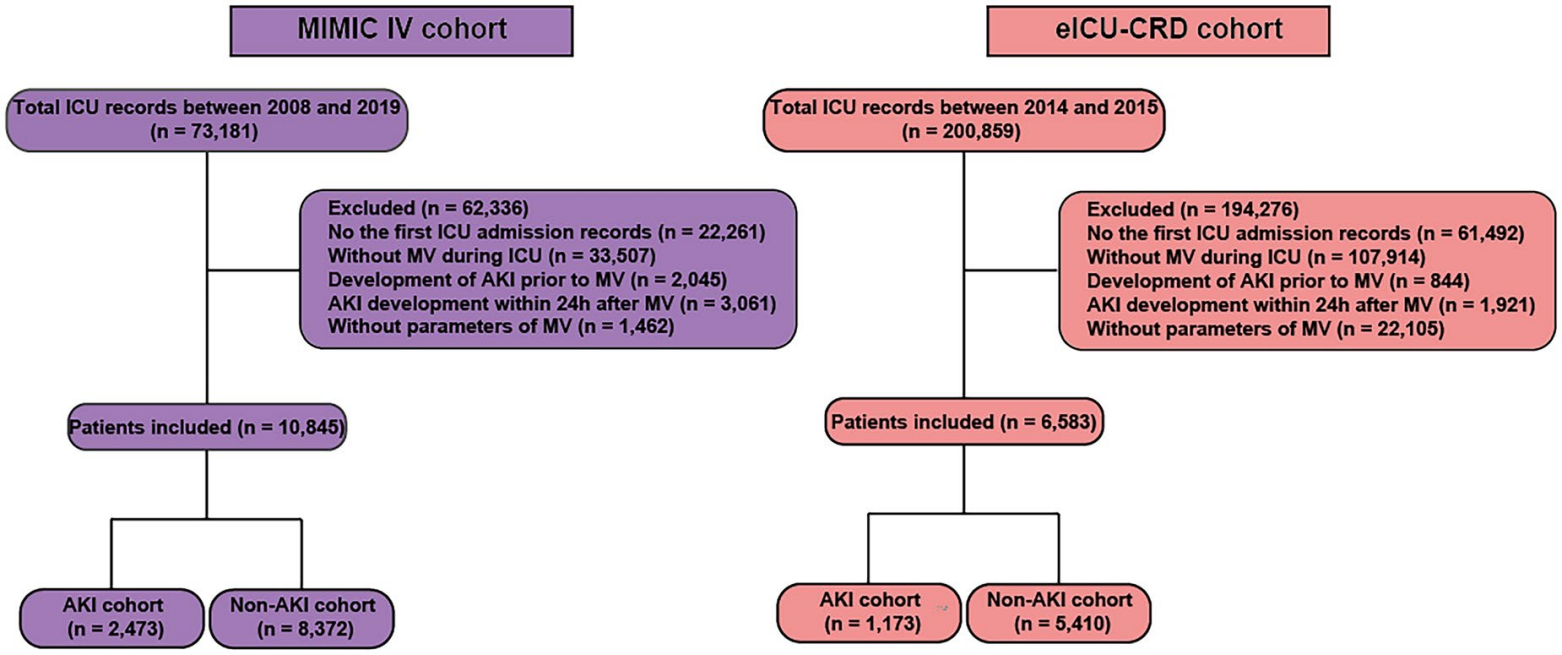
Flow diagram of patient selection from the MIMIC-IV and eICU-CRD cohorts.

During the study period, a total of 274,040 patients underwent initial screening. After exclusions, a final cohort of 17,428 patients was included in the analysis. The excluded patients consisted of 149,292 individuals who either did not receive mechanical ventilation, developed AKI prior to mechanical ventilation, or developed AKI within 24 h of initiating mechanical ventilation, as well as 107,320 patients who were non-first-time ICU admissions or had missing mechanical ventilation data. This final cohort comprised 13,782 AKI patients and 3,646 non-AKI patients (Figure 1).

In the MIMIC IV cohort, 10,845 patients were enrolled, with 8,372 in the non-AKI group and 2,473 (22.8%) in the AKI group. AKI patients were older than their non-AKI counterparts (*p <* 0.001). The proportions of coronary heart disease, heart failure, atrial fibrillation, diabetes mellitus, chronic kidney disease, liver disease, chronic pulmonary disease (all *p <* 0.001), and hypertension (*p =* 0.007) were higher in AKI patients. Both SOFA and APSIII scores were elevated in the AKI group (both *p <* 0.001). The AKI group had significantly decreased P/F ratio and PaCO_2_ levels (all *p* < 0.001). They also showed higher values of P_mean_, driving pressure, peak pressure, plateau pressure, PEEP, tidal volume, dynamic compliance, and mechanical power (all *p <* 0.001). AKI was linked to higher rates of CRRT, hospital mortality, ICU mortality, 28-day mortality, and 90-day mortality (all *p <* 0.001). In terms of laboratory findings, AKI patients had higher white blood cell counts, potassium, anion gap, lactate, and glucose (all *p <* 0.001), yet lower hemoglobin and platelets (all *p <* 0.001). Additionally, AKI patients had lower blood pressure and received more vasoactive drugs and diuretics (all *p <* 0.001) (Table 1).

**Table 1.** Baseline characteristics of patients with and without developing AKI.

	MIMIC IV			eICU-CRD		
	Non-AKI	AKI	*P*-value	Non-AKI	AKI	*P*-value
Number	8372	2473		5410	1173	
Age, years, M (Q1, Q3)	65 (53, 75)	70 (58, 79)	<0.001	65.00 (53, 75)	63 (51, 73)	0.001
Male, *n* (%)	5022 (60.0)	1429 (57.8)	0.053	3071 (56.8)	645 (55.0)	0.28
Body weight, Kg, M (Q1, Q3)	80.0 (67.4, 93.8)	80.0 (67.0, 95.0)	0.390	81.2 (67.5, 97.1)	83.2 (68.5, 101.2)	0.005
Complication, *n* (%)						
Coronary heart disease	2826 (33.8)	976 (39.5)	<0.001	890 (16.5)	169 (14.4)	0.092
Heart failure	1392 (16.6)	784 (31.7)	<0.001	597 (11.0)	144 (12.3)	0.243
Hypertension	4145 (49.5)	1147 (46.4)	0.007	1603 (29.6)	420 (35.8)	<0.001
Atrial fibrillation	2173 (26.0)	964 (39.0)	<0.001	409 (7.6)	92 (7.8)	0.787
Diabetes mellites	2038 (24.3)	815 (33.0)	<0.001	1359 (25.1)	381 (32.5)	<0.001
Chronic kidney disease	721 (8.6)	482 (19.5)	<0.001	274 (5.1)	135 (11.5)	<0.001
Liver disease	664 (7.9)	318 (12.9)	<0.001	189 (3.5)	50 (4.3)	0.234
Chronic Pulmonary disease	1824 (21.8)	690 (27.9)	<0.001	949 (17.5)	234 (19.9)	0.057
Anemia	3270 (39.1)	1295 (52.4)	<0.001	213 (3.9)	51 (4.3)	0.570
Malignant cancer	762 (9.1)	215 (8.7)	0.560	603 (11.1)	115 (9.8)	0.199
Kidney transplant	37 (0.4)	19 (0.8)	0.047	10 (0.2)	5 (0.4)	0.116
Laboratory parameter						
WBC, K/UL, M (Q_1_, Q_3_)	13.1 (9.9, 16.6)	13.3 (10.3, 17.7)	<0.001	13.7 (10.1, 17.2)	13.9 (10.7, 18.8)	<0.001
Hemoglobin, mg/dL, M (Q_1_, Q_3_)	10.3 (9.1, 11.7)	9.8 (8.7, 11.2)	<0.001	10.3 (8.7, 11.9)	9.9 (8.3, 11.8)	<0.001
Platelet, K/UL, M (Q_1_, Q_3_)	158 (124, 204)	155 (112, 203)	<0.001	159 (121, 212)	159 (115, 216)	0.482
MCV, dL, M (Q_1_, Q_3_)	14.1 (13.3, 15.0)	14.4 (13.7, 15.7)	<0.001	14.8 (13.8, 15.5)	15.1 (14.1, 16.1)	<0.001
RDW, %, M (Q_1_, Q_3_)	90 (87, 93)	90 (86, 93)	0.196	89 (87, 93)	90 (86, 94)	0.014
Na+, mmol/L, M (Q_1_, Q_3_)	140 (138, 142)	140 (138, 143)	0.001	141 (139, 144)	142 (139, 145)	0.075
K+, mmol/L, M (Q_1_, Q_3_)	4.4 (4.0, 4.7)	4.4 (4.1, 4.8)	<0.001	4.4 (4.0, 4.7)	4.4 (4.1, 4.9)	<0.001
AG, mmol/L, M (Q_1_, Q_3_)	14 (12, 16)	14 (12, 17)	<0.001	11 (9, 13.30)	13 (11, 16)	<0.001
P/F ratio, M (Q1, Q3)	231 (147,316)	170 (107,246)	<0.001	168 (110,228)	152 (103,209)	<0.001
PaCO2, mmHg, M (Q1, Q3)	38 (33,42)	35 (31,40)	<0.001	44 (40,49)	44 (39,50)	0.578
Lactate, mmol/L, M (Q1, Q3)	2.2 (1.6, 3.0)	2.3 (1.7, 3.4)	<0.001	2.7 (2.2, 2.9)	2.7 (2.0, 3.4)	<0.001
Glucose, mg/dL, M (Q1, Q3)	146 (125, 167)	152 (131, 178)	<0.001	174 (141, 212)	188 (150, 228)	<0.001
BUN, mg/dL, M (Q1, Q3)	16 (12, 20)	17 (14, 24)	<0.001	19 (14, 26)	26 (19, 39)	<0.001
Creatinine, mg/dL, M (Q1, Q3)	0.9 (0.7, 1.0)	0.9 (0.8, 1.1)	<0.001	1.0 (0.8, 1.2)	1.3 (1.0, 1.9)	<0.001
MAP_mean_, mmHg, M (Q1, Q3)	75.8 (71.0, 81.8)	74.7 (69.8, 80.4)	<0.001	76.7 (70.9,83.5)	76.7 (70.9,83.6)	0.999
MAPmin, mmHg, M (Q1, Q3)	59 (53, 65)	57 (52, 63)	<0.001	56 (49, 63)	54 (47, 62)	<0.001
I/O balance, ml, M (Q1, Q3)	1281.12 (110.30, 2605.69)	1998.54 (628.47, 3689.75)	<0.001	−327.46 (−1324.38, 1066.50)	−462.62 (−1603.77, 1030.00)	0.013
Intervention, *n* (%)						
Vasoactive drugs use	2085 (24.9)	1015 (41.0)	<0.001	2189 (40.5)	599 (51.1)	<0.001
Nephrotoxic drug exposure	3883 (46.38)	1440 (58.23)	<0.001	485 (8.96)	102 (8.70)	0.769
Diuretics exposure	2708 (32.35)	967 (39.10)	<0.001	602 (11.1)	100 (8.5)	0.01
MV parameter						
Peak pressure, cmH_2_O, M (Q1, Q3)	19 (16, 22)	21 (18, 25)	<0.001	22 (19, 26)	23 (20, 28)	<0.001
Plat pressure, cmH_2_O, M (Q1, Q3)	17 (15, 19)	18 (16, 21)	<0.001	18 (15, 21)	19 (16, 23)	<0.001
PEEP, cmH_2_O, M (Q1, Q3)	5 (5, 5)	5 (5, 8)	<0.001	5 (5, 6)	5 (5, 7)	<0.001
Tidal volume, ml, M (Q1, Q3)	485 (434, 536)	476 (424, 527)	<0.001	500 (450, 550)	500 (450, 550)	0.004
Respiratory rate, cpm, M (Q1, Q3)	18 (16, 20)	18 (16, 21)	<0.001	18 (15, 21)	19 (16, 23)	<0.001
Mean airway pressure, cmH_2_O, M (Q1, Q3)	8.6 (7.8,10.0)	9.3 (8.1,11.9)	<0.001	9.8 (8.7, 11.7)	10.8 (9.3, 12.9)	<0.001
Driving pressure, cmH_2_O, M (Q1, Q3)	11 (9, 13)	12 (10, 14)	<0.001	12 (10, 15)	13 (10, 16)	<0.001
Dynamic driving pressure, cmH_2_O, M (Q1, Q3)	14 (11, 16)	15 (12, 18)	<0.001	16 (13, 20)	17 (14, 21)	<0.001
Dynamic compliance, M (Q1, Q3)	35.4 (28.8, 44.1)	32.3 (26.1, 40.6)	<0.001	31.6 (25.0, 40.0)	29.4 (23.8, 36.7)	<0.001
Minute ventilation, L, M (Q1, Q3)	8.7 (7.6, 9.9)	8.8 (7.6, 10.2)	0.007	9.0 (7.7, 10.5)	9.5 (8.1, 11.2)	<0.001
Mechanical power, J/min, M (Q1, Q3)	11.3 (8.8, 14.4)	12.6 (9.6, 16.5)	<0.001	13.6 (10.6, 18.0)	15.4 (11.8, 20.5)	<0.001
Disease severity score						
SOFA score, M (Q_1_, Q_3_)	5 (3, 7)	7 (5, 10)	<0.001	8 (6, 10)	9 (7, 12)	<0.001
APSIII score, M (Q_1_, Q_3_)	37 (27, 55)	56 (39, 79)	<0.001			
APACHEII score, M (Q_1_, Q_3_)				75 (58, 90)	82 (70, 106)	<0.001
ICU department, *n* (%)			<0.001			<0.001
MICU/CCU	4630 (55.30)	1559 (63.04)		749 (13.85)	185 (15.78)	
Med-SICU	763 (9.11)	237 (9.58)		3154 (58.33)	781 (66.64)	
SICU	2979 (35.58)	677 (27.38)		1504 (27.82)	206 (17.58)	
Outcomes						
CRRT after MV, *n* (%)	40 (0.5)	144 (5.8)	<0.001	145 (2.7)	180 (15.3)	<0.001
Hospital mortality, *n* (%)	953 (11.4)	526 (21.3)	<0.001	812 (15.0)	164 (14.0)	0.394
ICU mortality, *n* (%)	746 (8.9)	438 (17.7)	<0.001	1011 (18.7)	226 (19.3)	0.675
28-day mortality, *n* (%)	1101 (13.2)	541 (21.9)	<0.001			
90-day mortality, *n* (%)	1341 (16.0)	676 (27.3)	<0.001			

Abbreviation: WBC: white blood cell; MCV, mean corpuscular volume; RDW, red cell distribution width; P/F ratio, Oxygenation index; AG, anion gap; BUN, blood urea nitrogen; MAP: Mean arterial pressure; SOFA, Sequential organ failure assessment; APS, acute physiology score; SAPS, simplified acute physiology score; APACHE II: Acute Physiology and Chronic Health Evaluation II; CRRT, continuous renal replacement therapy; MV, mechanical ventilation.

For the eICU – CRD cohort, 6,583 patients were included, including 5,410 in the non-AKI group and 1,173 (17.8%) in the AKI group. AKI patients had a higher body weight (*p =* 0.005), while age (*p =* 0.001) and gender distribution (*p =* 0.28) were comparable between the two groups. The AKI group had higher proportions of hypertension, diabetes mellitus, and chronic kidney disease. But no significant difference was found in chronic pulmonary disease (*p =* 0.057). The AKI group had significantly decreased P/F ratio (*p* < 0.001), but not in PaCO2 levels (*p* = 0.578). SOFA scores and APACHE II scores were higher in AKI patients (both *p <* 0.001). AKI was associated with a higher CRRT rate (*p <* 0.001), whereas hospital mortality (*p =* 0.394) and ICU mortality (*p =* 0.675) were similar between the groups. AKI patients exhibited higher peak pressure, plateau pressure, PEEP, P_mean_, and mechanical power (all *p <* 0.001). They had lower blood pressure, received more vasoactive drugs (*p <* 0.001) but fewer diuretics (all *p <* 0.001). Laboratory results showed that AKI patients had higher potassium, anion gap, lactate, glucose, blood urea nitrogen, and creatinine, but lower hemoglobin (all *p <* 0.001) (Table 1).

### 3.2. Multimodal feature selection and variable importance analysis in MIMIC IV and eICU cohorts

**Figure 2.**
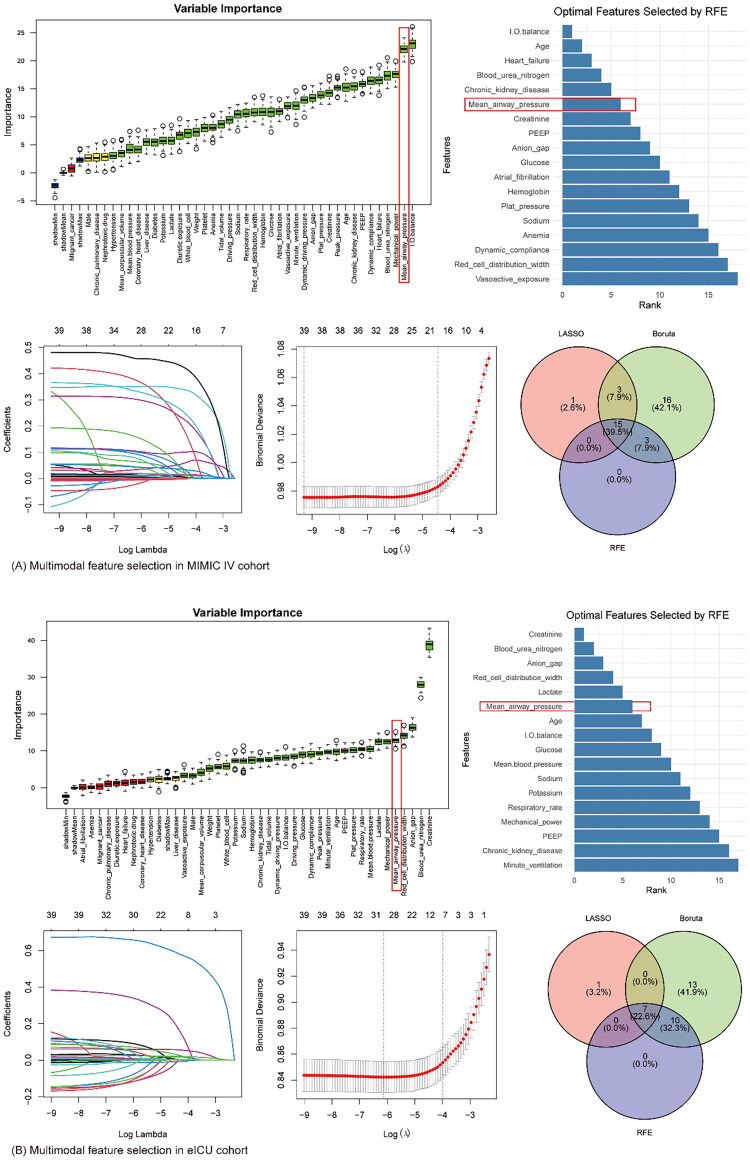
Multimodal feature selection and variable importance analysis. Core variables were selected using a combination of the Boruta algorithm, recursive feature elimination, and LASSO regression with 10-fold cross-validation. The outcomes from these methods were integrated via a Venn diagram, with the intersection representing the final set of key variables. (A) Multimodal Feature Selection in MIMIC-IV cohort. (B) Multimodal Feature Selection in eICU cohort.

Multimodal feature selection employed three algorithms: Recursive Feature Elimination (RFE), Random Forest, and LASSO regression. It aimed to identify robust biomarkers associated with AKI in mechanically ventilated patients (Figure 2). The intersection of all the features prioritized by the three algorithms in the two databases includes P_mean_, age, BUN and SOFA scores.

P_mean_ emerged as the most prominent shared biomarker, consistently ranked as one of the top features across both cohorts. In RFE analysis, P_mean_ exhibited the one of the top 7 highest importance score, with a significant reduction in binomial deviance when included in predictive models. Random Forest further validated its relevance through high Gini importance, and LASSO regression retained P_mean_ in the final parsimonious model with non-zero coefficients, confirming its independent predictive value in both cohorts.

### 3.3. Clinical outcomes of patients in different categories of P_mean_

Patients were stratified into quartiles based on P_mean_ values from the MIMIC IV cohort (Q1: ≤7.8 cmH_2_O; Q2: 7.8–8.7 cmH_2_O; Q3: 8.7–10.3 cmH_2_O; Q4: >10.3 cmH_2_O). Table 2 demonstrates a consistent, graded association between increasing P_mean_ quartiles and worsening clinical outcomes in both cohorts (all *P*-values for trend <0.001) (Table 2).

**Table 2.** Clinical outcomes of patients in different categories of mean airway pressure.

	Q1 (P_mean_≤7.8)	Q2 (7.8 < P_mean_≤8.7)	Q3 (8.7 < P_mean_≤10.3)	Q4 (P_mean_>10.3)	*P*-value
MIMIC IV cohort					
Number	2405	3116	2533	2791	
Developing AKI, *n* (%)	376 (15.63)	569 (18.26)	607 (23.96)	921 (33.00)	<0.001
AKI Stage, *n* (%)					<0.001
Stage 1	295 (78.46)	428 (75.22)	437 (71.99)	562 (61.02)	
Stage 2	55 (14.63)	91 (15.99)	109 (17.96)	187 (20.30)	
Stage 3	26 (6.91)	50 (8.79)	61 (10.05)	172 (18.68)	
Developing AKI in survivals, *n* (%)	313 (14.50)	469 (16.83)	490 (22.24)	675 (30.45)	<0.001
CRRT after MV, *n* (%)	9 (0.37)	19 (0.61)	33 (1.30)	123 (4.41)	<0.001
Hospital mortality, *n* (%)	246 (10.23)	329 (10.56)	330 (13.03)	574 (20.57)	<0.001
ICU mortality, *n* (%)	152 (6.32)	247 (7.93)	269 (10.62)	516 (18.49)	<0.001
28-day mortality, *n* (%)	289 (12.02)	387 (12.42)	369 (14.57)	597 (21.39)	<0.001
90-day mortality, *n* (%)	384 (15.97)	476 (15.28)	452 (17.84)	705 (25.26)	<0.001
Hospital LOS, day, M (Q_1_, Q_3_)	7.5 (5.1,12.3)	7.2 (4.9,11.8)	7.9 (5.2,12.9)	9.3 (5.6,15.9)	<0.001
ICU LOS, day, M (Q_1_, Q_3_)	2.4 (1.4,4.7)	2.3 (1.3,4.7)	3.0 (1.5,5.8)	4.5 (2.2,9.1)	<0.001
eICU cohort					
Number	448	1145	2000	2990	
Developing AKI, *n* (%)	49 (10.94)	127 (11.09)	317 (15.85)	680 (22.74)	<0.001
AKI Stage, *n* (%)					0.015
Stage 1	35 (71.43)	90 (70.87)	204 (64.35)	390 (57.35)	
Stage 2	5 (10.20)	18 (14.17)	37 (11.67)	95 (13.97)	
Stage 3	9 (18.37)	19 (14.96)	76 (23.97)	195 (28.68)	
Developing AKI in survivals, *n* (%)	44 (11.03)	112 (10.92)	271 (15.58)	520 (23.83)	<0.001
CRRT after MV, *n* (%)	11 (2.46)	22 (1.92)	78 (3.90)	214 (7.16)	<0.001
Hospital mortality, *n* (%)	49 (10.94)	119 (10.39)	261 (13.05)	808 (27.02)	<0.001
ICU mortality, *n* (%)	37 (8.26)	87 (7.60)	178 (8.90)	674 (22.54)	<0.001
Hospital LOS, day, M (Q_1_, Q_3_)	6.1 (4.0,10.2)	6.4 (3.9,10.8)	7.3 (4.4,12.2)	8.2 (4.5,14.8)	<0.001
ICU LOS, day, M (Q_1_, Q_3_)	2.1 (1.2,4.0)	2.3 (1.2,4.3)	2.7 (1.7,5.0)	3.7 (1.8,7.8)	<0.001

Abbreviation: P_mean_, mean airway pressure; CRRT, continuous renal replacement therapy; ICU, intensive care unit; LOS, length of stay.

In the MIMIC IV cohort, we observed a progressive increase in AKI incidence from 15.6% in Q1 to 33.0% in Q4, with a corresponding rise in severe AKI cases (Stage 3) from 6.9% to 18.7%. This pattern persisted among survivors (14.5% to 30.5%) and was accompanied by a marked increase in CRRT utilization (0.4% to 4.4%). Mortality outcomes showed similar deterioration, with ICU mortality tripling from 6.3% to 18.5% across quartiles. Both hospital (7.5 to 9.3 days) and ICU (2.4 to 4.5 days) length of stay significantly lengthened with higher P_mean_ levels.

The eICU-CRD cohort replicated these findings, with AKI incidence doubling from 10.9% to 22.7% and Stage 3 AKI increasing from 18.4% to 28.7%. CRRT use rose from 2.5% to 7.2%, while ICU mortality increased nearly threefold (8.3% to 22.5%). Hospital and ICU stay progressively extended from 6.1 to 8.2 days and 2.1 to 3.7 days, respectively, across quartiles.

### 3.4. Logistic regression analysis evaluating the predictive of P_mean_ on AKI development

Logistic regression analyses revealed a significant dose-response relationship between elevated P_mean_ and AKI risk in both cohorts (Table 3). Each 1-cmH_2_O increase in P_mean_ was independently associated with higher AKI odds after sequential adjustments: in the fully adjusted model (Model-3), which included demographics, comorbidities, severity scores, interventions, dynamic compliance, P/F ratio, PaCO_2_, and chronic pulmonary disease, the OR was 1.05 (95% CI 1.03–1.07, *p* < 0.001) for MIMIC-IV and 1.06 (95% CI 1.04–1.09, *p <* 0.001) for eICU-CRD.

**Table 3.** Logistic regression analysis evaluating the predictive of mean airway pressure on AKI development.

	Unadjusted model		Adjusted model-1		Adjusted model-2		Adjusted model-3	
	OR (95%CI)	*P-*value	OR (95%CI)	*P-*value	OR (95%CI)	*P-*value	OR (95%CI)	*P-*value
MIMIC IV cohort								
Mean airway pressure, cmH_2_O	1.15 (1.13–1.17)	*<0.001*	1.17 (1.15–1.19)	*<0.001*	1.15 (1.13–1.17)	*<0.001*	1.05 (1.03–1.07)	*<0.001*
Categories								
Q1 (P_mean_≤7.8)	Reference		Reference		Reference		Reference	
Q2 (7.8 < P_mean_≤8.7)	1.21 (1.05–1.39)	0.010	1.22 (1.05–1.40)	0.007	1.21 (1.04–1.40)	0.013	1.11 (0.95–1.29)	0.174
Q3 (8.7 < P_mean_≤10.3)	1.70 (1.47–1.96)	<0.001	1.73 (1.49–1.99)	<0.001	1.61 (1.38–1.86)	<0.001	1.25 (1.07–1.46)	0.004
Q4 (P_mean_>10.3)	2.66 (2.32–3.04)	<0.001	2.84 (2.47–3.27)	<0.001	2.53 (2.19–2.92)	<0.001	1.37 (1.17–1.60)	<0.001
eICU cohort								
Mean airway pressure, cmH_2_O	1.10 (1.08–1.12)	<0.001	1.10 (1.08–1.12)	<0.001	1.10 (1.08–1.12)	<0.001	1.06 (1.04–1.09)	<0.001
Categories								
Q1 (P_mean_≤7.8)	Reference		Reference		Reference		Reference	
Q2 (7.8 < P_mean_≤8.7)	1.02 (0.72–1.44)	0.930	1.02 (0.72–1.45)	0.914	1.05 (0.41–1.49)	0.792	1.04 (0.73–1.49)	0.813
Q3 (8.7 < P_mean_≤10.3)	1.53 (1.11–2.11)	0.009	1.55 (1.12–2.13)	0.008	1.54 (1.12–2.13)	0.008	1.47 (1.06–2.04)	0.021
Q4 (P_mean_>10.3)	2.40 (1.76–3.26)	<0.001	2.40 (1.76–3.28)	<0.001	2.39 (1.75–3.27)	<0.001	1.96 (1.43–2.70)	<0.001

Adjusted model-1: Demographic information was adjusted.

Adjusted model-2 Further adjustments for comorbidities were performed based on the adjusted model-1.

Adjusted model-3 Further adjustments for specific interventions, disease severity scores, dynamic compliance, PaO₂/FiO ratio₂, PaCO₂, and chronic pulmonary disease were performed based on the adjusted model-2.

Critical risk stratification was observed across quartiles: patients with P_mean_ >10.3 cmH_2_O (Q4) exhibited 37% higher AKI risk in MIMIC-IV (aOR 1.37, 95% CI 1.17–1.60, *p <* 0.001) and 96% higher risk in eICU-CRD (aOR 1.96, 1.43–2.70, *p <* 0.001) compared to the lowest quartile (Q1: ≤7.8 cmH_2_O). The intermediate quartile (Q3: 8.7–10.3 cmH_2_O) also demonstrated significant risk elevation (MIMIC-IV: aOR 1.25, 1.07–1.46, *p* = 0.004; eICU-CRD: aOR 1.47, 1.06–2.04, *p* = 0.021), whereas the Q2 group (7.8–8.7 cmH_2_O) showed no statistically significant increase after full adjustment (MIMIC-IV: aOR 1.11, 0.95–1.29, *p* = 0.174; eICU-CRD: aOR 1.05, 0.73–1.49, *p* = 0.813).

### 3.5. Non-linear relationships between P_mean_ and clinical outcomes

**Figure 3.**
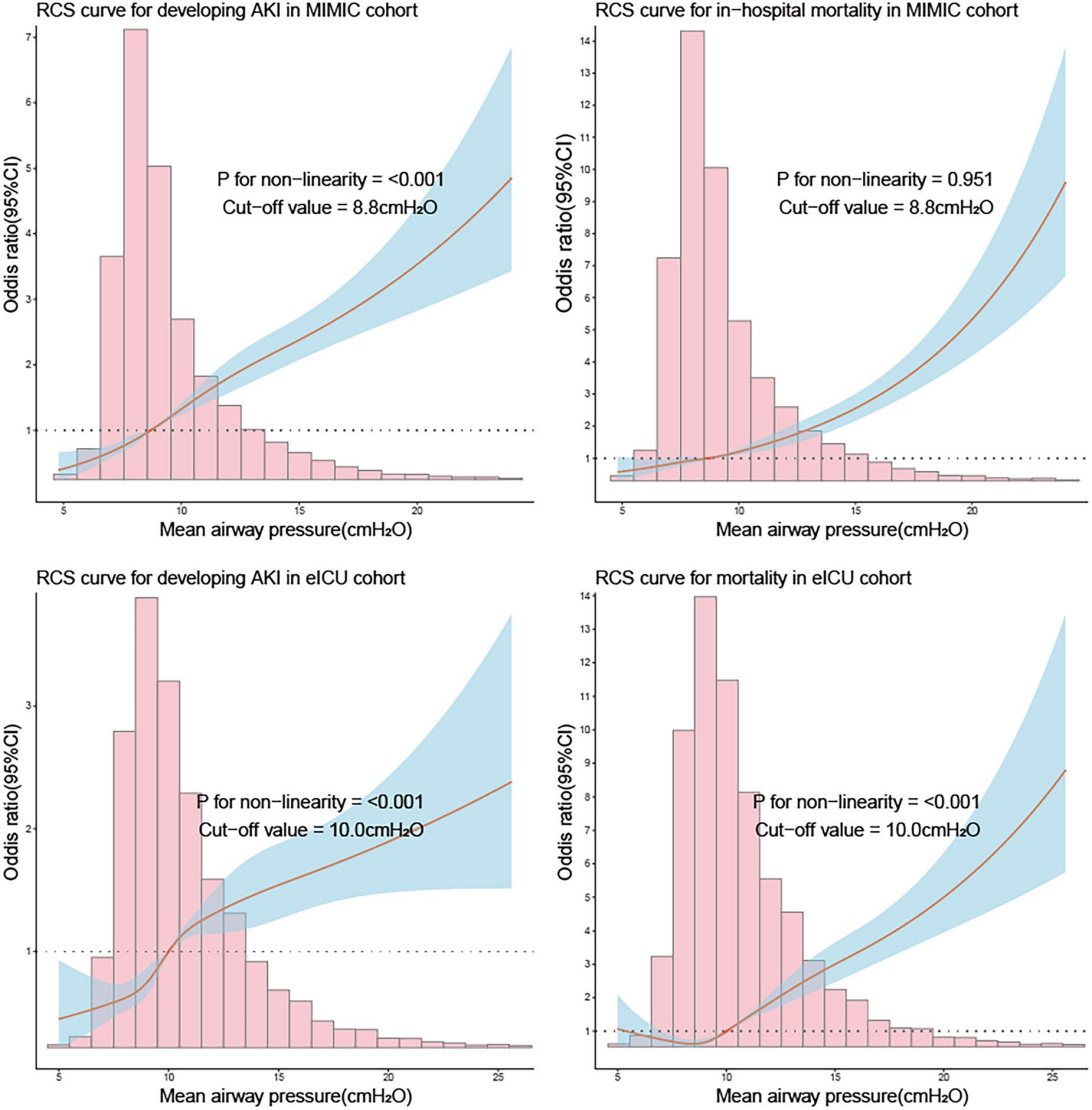
Non-linear relationships between P_mean_ and clinical outcomes. (A) RCS curve for developing AKI in MIMIC-IV cohort. (B) RCS curve for developing AKI in eICU-CRD cohort. (C) RCS curve for mortality in MIMIC-IV cohort. (D) RCS curve for mortality in eICU-CRD cohort.

RCS analyses showed the non-linear relationships (*P* for non-linearity < 0.001) between P_mean_ and the risk of developing AKI in mechanically ventilated patients from the MIMIC-IV and eICU-CRD cohorts (Figure 3). In the MIMIC-IV cohort, the AKI risk curve exhibited a J-shaped relationship with an inflection point at 8.8 cmH_2_O (Figure 3A). Similarly, the eICU-CRD cohort showed a non-linear dose-response relationship with a higher threshold at 10.0 cmH_2_O (Figure 3B). Below these cutoff values, the association was attenuated and the curve suggested a lower observed AKI risk, while above these thresholds, each cmH2O increase was associated with progressively higher AKI risk.

Both cohorts showed a positive correlation between P_mean_ and mortality risk. The MIMIC-IV cohort displayed a linear association (*P* for non-linearity = 0.951) with P_mean_ (Figure 3C). In contrast, the eICU-CRD cohort revealed a significant non-linear relationship (*P* for non-linearity <0.001) with a critical threshold at 10.0 cmH_2_O (Figure 3D), where P_mean_ values exceeding this point were associated with substantially increased mortality risk. The non-linear relationship between initial airway pressure and prognostic risk remained consistent, with both AKI risk and in-hospital mortality risk showing an increasing trend as initial airway pressure rose (as shown in Figure S1).

### 3.6. Result in the PSM cohort

Based on the cutoff value obtained from the RCS, patients were categorized into low-P_mean_ and high-P_mean_ groups. Through propensity score matching (PSM), we effectively balanced the baseline characteristics between the two groups, enhancing comparability (Table 4). In the PSM cohort, we demonstrated significantly worse clinical outcomes in patients with elevated P_mean_ (>8.7 cmH_2_O) compared to those with lower P_mean_ (≤8.7 cmH_2_O) in both cohorts (Table 4). In the MIMIC-IV cohort (*n* = 3,389 matched pairs), the high-P_mean_ group exhibited higher rates of AKI (22.9% vs 20.2%, *p* = 0.005), hospital mortality (14.2% vs 10.8%, *p <* 0.001), and ICU mortality (11.9% vs 7.8%, *p <* 0.001), along with prolonged ICU length of stay (median 3.0 vs 2.6 days, *p <* 0.001). The eICU-CRD cohort (*n* = 1,545 matched pairs) showed similar patterns, with the high-P_mean_ group displaying increased AKI incidence (15.7% vs 11.1%, *p <* 0.001), higher hospital mortality (13.9% vs 10.6%, *p* = 0.006), and longer ICU stays (median 3.0 vs 2.0 days, *p <* 0.001).

**Table 4.** Comparison for patients with low-P_mean_ and high-P_mean_ in the PSM cohort.

	MIMIC IV cohort			eICU cohort		
	low-P_mean_	high-P_mean_	*P*-value	low-P_mean_	high-P_mean_	*P*-value
Number	3389	3389		1545	1545	
Age, years, M (Q1, Q3)	67 (55, 77)	66 (55, 77)	0.95	64 (50, 75)	63 (49, 75)	0.82
Male, *n* (%)	2030 (59.9)	2045 (60.3)	0.728	876 (56.7)	903 (58.4)	0.344
Body weight, Kg, M (Q1, Q3)	79.5 (67.7, 92.4)	79.3 (67.0, 91.4)	0.213	75.9 (63.6, 89.3)	73.6 (62.8, 86.2)	0.003
Complication, *n* (%)						
Coronary heart disease	1248 (36.8)	1267 (37.4)	0.651	216 (14.0)	208 (13.5)	0.714
Heart failure	676 (19.9)	619 (18.3)	0.084	122 (7.9)	87 (5.6)	0.015
Hypertension	1691 (49.9)	1676 (49.5)	0.734	439 (28.4)	439 (28.4)	1.000
Atrial fibrillation	1002 (29.6)	1007 (29.7)	0.915	105 (6.8)	97 (6.3)	0.610
Diabetes mellites	886 (26.1)	868 (25.6)	0.637	308 (19.9)	253 (16.4)	0.012
Chronic kidney disease	378 (11.2)	376 (11.1)	0.969	78 (5.0)	66 (4.3)	0.348
Liver disease	303 (8.9)	293 (8.6)	0.699	61 (3.9)	70 (4.5)	0.475
Chronic Pulmonary disease	784 (23.1)	743 (21.9)	0.245	182 (11.8)	158 (10.2)	0.186
Anemia	1437 (42.4)	1446 (42.7)	0.844	62 (4.0)	74 (4.8)	0.335
Malignant cancer	307 (9.1)	318 (9.4)	0.675	159 (10.3)	147 (9.5)	0.508
Kidney transplant	18 (0.5)	16 (0.5)	0.863	3 (0.2)	4 (0.3)	>0.999
I/O balance, ml, M (Q1, Q3)	1379 (211, 2798)	1418 (201, 2805)	0.879	−327 (−1389, 999)	−349 (−1392, 1065)	0.934
Intervention, *n* (%)						
Vasoactive drugs use	911 (26.9)	854 (25.2)	0.121	484 (31.3)	380 (24.6)	<0.001
Nephrotoxic drug exposure	1654 (48.8)	1625 (47.9)	0.496	151 (9.8)	157 (10.2)	0.764
Diuretics exposure	1187 (35.0)	1180 (34.8)	0.878	119 (7.7)	101 (6.5)	0.234
Disease severity score						
SOFA score, M (Q_1_, Q_3_)	5 (4, 8)	5 (4, 7)	0.144	8 (6, 9)	7 (5, 9)	0.002
APSIII score, M (Q_1_, Q_3_)	40 (28, 59)	39 (28, 57)	0.150			
APACHEII score, M (Q_1_, Q_3_)				74 (56, 84)	71 (52, 80)	0.004
Blood gas partial pressure						
P/F ratio, mmHg, M (Q1, Q3)	216 (143, 289)	213 (143, 283)	0.702	209 (147, 269)	209 (160, 296)	<0.001
PaCO2, mmHg, M (Q1, Q3)	37 (33, 41)	37 (33, 41)	0.525	43 (39, 46)	43 (37, 46)	0.002
MV parameter						
Peak pressure, cmH_2_O, M (Q1, Q3)	17 (15, 19)	22 (20, 25)	<0.001	18 (16, 20)	22 (19, 25)	<0.001
Plat pressure, cmH_2_O, M (Q1, Q3)	16 (14, 17)	19 (17, 21)	<0.001	15 (13, 17)	18 (15, 20)	<0.001
PEEP, cmH_2_O, M (Q1, Q3)	5 (5, 5)	5 (5, 7)	<0.001	5 (5, 5)	5 (5, 5)	<0.001
Tidal volume, ml, M (Q1, Q3)	486 (434, 539)	482 (432, 532)	0.011	500 (450, 543)	500 (450, 550)	0.001
Respiratory rate, cpm, M (Q1, Q3)	17 (16, 19)	18 (17, 21)	<0.001	16 (14, 18)	18 (15, 20)	<0.001
Mean airway pressure, cmH_2_O, M (Q1, Q3)	8.0 (7.4, 8.3)	9.9 (9.2, 11.3)	<0.001	8.1 (7.7, 8.4)	10.0 (9.3, 11.2)	<0.001
Driving pressure, cmH_2_O, M (Q1, Q3)	11 (9, 13)	12 (11, 15)	<0.001	10 (8, 12)	12 (10, 15)	<0.001
Dynamic driving pressure, cmH_2_O, M (Q1, Q3)	12 (10, 14)	16 (13, 18)	<0.001	13 (11, 15)	16 (14, 19)	<0.001
Dynamic compliance, M (Q1, Q3)	39.6 (33.3, 48.6)	30.2 (25.1, 36.5)	<0.001	38.5 (31.6, 45.8)	30.7 (25.0, 37.5)	<0.001
Minute ventilation, L, M (Q1, Q3)	8.5 (7.5, 9.6)	8.9 (7.8, 10.1)	<0.001	8.0 (6.8, 9.0)	8.8 (7.7, 10.0)	<0.001
Mechanical power, J/min, M (Q1, Q3)	9.6 (7.9, 11.5)	13.5 (11.4, 16.4)	<0.001	9.9 (8.1, 12.2)	13.7 (11.1, 16.9)	<0.001
Outcomes						
Developing AKI, *n* (%)	683 (20.2)	777 (22.9)	0.005	172 (11.1)	243 (15.7)	<0.001
Developing AKI in survivals, *n* (%)	558 (18.5)	627 (21.6)	0.003	152 (11.0)	213 (16.0)	<0.001
CRRT after MV, *n* (%)	27 (0.8)	38 (1.1)	0.213	33 (2.1)	47 (3.0)	0.141
Hospital mortality, *n* (%)	367 (10.8)	480 (14.2)	<0.001	164 (10.6)	215 (13.9)	0.006
ICU mortality, *n* (%)	265 (7.8)	402 (11.9)	<0.001	121 (7.8)	149 (9.6)	0.085
28-day mortality, *n* (%)	423 (12.5)	527 (15.6)	<0.001			
90-day mortality, *n* (%)	542 (16.0)	644 (19.0)	0.001			
Hospital LOS, day, M (Q_1_, Q_3_)	7.7 (5.2, 12.5)	7.8 (5.1, 12.7)	0.824	6.3 (4.0, 10.7)	7.4 (4.3, 12.9)	<0.001
ICU LOS, day, M (Q_1_, Q_3_)	2.6 (1.4, 5.0)	3.0 (1.5, 6.0)	<0.001	2.2 (1.3, 4.2)	3.0 (1.7, 6.2)	<0.001

Abbreviation: P_mean_, Mean airway pressure; SOFA, Sequential Organ Failure Assessment; APS III, Acute Physiology Score III; APACHE II, Acute Physiology and Chronic Health Evaluation II; PEEP, Positive End-Expiratory Pressure; MV, Mechanical Ventilation; CRRT, Continuous Renal Replacement Therapy; ICU, Intensive Care Unit; LOS, Length of Stay.

### 3.7. Subgroup analysis

**Figure 4.**
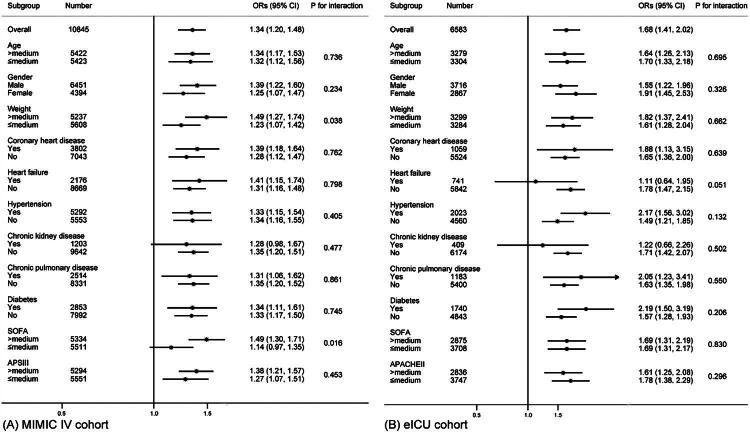
Subgroup analysis of the association between P_mean_ and AKI risk. (A) Subgroup analysis of the association between P_mean_ and AKI risk in MIMIC-IV cohort. (B) Subgroup analysis of the association between P_mean_ and AKI risk in eICU-CRD cohort.

Figure 4 presents the result of subgroup analyses in the MIMIC-IV (Panel A) and eICU-CRD (Panel B) cohorts. In the MIMIC-IV cohort, elevated P_mean_ was associated with increased AKI risk across all subgroups (all ORs > 1). Aside from a significant weight × P_mean_ interaction (*P* for interaction = 0.047) and SOFA × P_mean_ interaction (*P* for interaction = 0.022) in MIMIC cohort, no other notable interactions were observed (all *P* for interaction > 0.05). The ORs for mortality were 1.35 (95% CI: 1.15–1.58) in patients with higher body weight and 1.14 (95% CI: 0.98–1.32) in patients with lower body weight. Among patients with higher SOFA scores, elevated P_mean_ had a more pronounced effect on the outcome, with an OR of 1.37 (95% CI: 1.19–1.57), significantly higher than that in the low SOFA group (OR: 1.05, 95% CI: 0.89–1.25). However, this difference was not observed in the APSIII subgroup (P for interaction = 0.580). The eICU-CRD cohort showed similar associations, with all ORs greater than 1. And no significant interactions were observed (all *P* for interaction > 0.05). Across ICU admission types, higher P_mean_ was consistently associated with increased AKI risk (all ORs > 1). Some associations did not reach statistical significance, likely due to smaller sample sizes; however, no significant interaction was observed (P for interaction > 0.05).

## 4. Discussion

In this multicenter retrospective cohort study, 1) elevated P_mean_ was independently associated with an increased risk of developing AKI. A clear non-linear dose–response relationship was observed, with threshold effects at approximately 8.8 cmH_2_O in the MIMIC-IV cohort and 10.0 cmH_2_O in the eICU-CRD cohort. The J-shaped pattern observed in the RCS analysis should be interpreted cautiously. The apparently lower-risk segment at lower P_mean_ may reflect case-mix differences and residual confounding, because patients with lower P_mean_ often require less ventilatory support and may have less severe lung disease and more stable physiology. Lower Pmean may also be more common during early improvement or weaning, and spline estimates at the lower tail can be influenced by sparse data and measurement variability. Therefore, we consider this non-linear pattern hypothesis-generating rather than evidence of a causal protective effect. 2) Elevated P_mean_ was further associated with a spectrum of adverse outcomes, including higher AKI severity, increased requirement for renal replacement therapy, elevated mortality, and prolonged ICU stay. Although absolute mortality differed between MIMIC-IV and eICU-CRD across some comparisons, mortality generally increased with higher P_mean_ in both cohorts, supporting the robustness of the exposure–outcome pattern across independent databases. 3) The association between elevated P_mean_ and AKI remained robust across multivariable regression, propensity score matching, and subgroup analyses, after comprehensive adjustment for illness severity (APS III, APACHE II, SOFA), pulmonary status (chronic pulmonary disease, P/F ratio, PaCO_2_, dynamic compliance), as well as vasoactive drug use, fluid balance, and nephrotoxic exposures.

Our findings extend and refine prior evidence linking elevated P_mean_ to AKI risk. While previous studies have reported this association in specific populations, such as cardiac surgery [14], pediatric ARDS [15], and abdominal surgery [16] patients, our multicenter analysis of two large, heterogeneous cohorts generalizes this risk across the critically ill spectrum. Importantly, our study extends prior evidence by identifying clinically relevant P_mean_ thresholds and proposing plausible hemodynamic and inflammatory pathways that may underlie the observed association between higher P_mean_ and AKI. A recent study by Dong et al. specifically investigated septic shock patients and innovatively demonstrated that dynamic P_mean_ trajectories over 72 h were strongly associated with mortality and AKI [17]. Our findings complement and extend this work by establishing the generalizability of P_mean_-associated renal risk across a broad, heterogeneous ICU population. Furthermore, we identified a clinically practical static threshold within the critical first 24 h, and through machine learning, positioned P_mean_ as the foremost ventilatory risk factor. Together, these studies support P_mean_ as a potential target for future kidney-protective ventilation strategies. Thus, as an integrative parameter reflecting cardiopulmonary load, P_mean_ may serve as a practical biomarker to guide ventilation optimization. These findings support future prospective studies to evaluate whether P_mean_-guided ventilation strategies can improve renal outcomes. The identification of a clinically relevant P_mean_ threshold may help generate hypotheses for renal-protective ventilation strategies and supports prospective evaluation beyond lung protection alone.

Mechanical ventilation is a well-established risk factor for AKI in critically ill patients [6,7], primarily through hemodynamic compromises induced by elevated airway pressures [8]. However, the relative importance of specific ventilatory parameters remains unclear. In this study, we focused on P_mean_ because it integrates peak inspiratory pressure, PEEP, and the inspiratory-to-expiratory ratio, thereby providing a more comprehensive reflection of the overall mechanical load and mean intrathoracic pressure than isolated parameters [9,10]. The fact that P_mean_ consistently emerged as the most influential ventilatory parameter in our machine learning analysis strongly validates this rationale and warrants investigation into the underlying pathophysiological pathways. The high P_mean_ groups in our study were associated with higher mechanical power and driving pressures, both of which have been implicated in ventilator-induced lung injury (VILI) and systemic inflammation. Prior studies suggest that inflammatory mediators such as IL-6 and TNF-α, released during biotrauma or alveolar overdistension, may directly contribute to renal tubular damage [18].

Our findings demonstrate that the association between elevated P_mean_ and AKI remained robust after comprehensive adjustment for severity scores, comorbidities, and interventions, suggesting an association beyond measured confounding. Prior studies have shown that modulation of P_mean_ during cardiopulmonary resuscitation or myocardial revascularization can significantly alter hemodynamic parameters, including cardiac index, cardiac output, stroke volume, and mean arterial pressure [14,19,20]. In another hand, elevated P_mean_ increases intrathoracic pressure, which reduces venous return and elevates central venous pressure (CVP), which may contribute to venous congestion [21–26]. Venous congestion may further reduce glomerular filtration pressure, elevate renal interstitial pressure, and impair tubular oxygen delivery, thereby creating a pathophysiological pathway linking elevated P_mean_ to AKI [22,27]. In our study, patients with higher P_mean_ exhibited significantly higher SOFA scores and required more vasoactive drug support, indicating substantial cardiopulmonary impairment. This is consistent with the findings of the mentioned studies.

Importantly, these two findings align with the concept of lung–kidney cross-talk [17,28], whereby VILI can propagate systemic inflammation, alter hemodynamics, and thereby exacerbate renal injury. This interaction highlights that AKI in mechanically ventilated patients may not solely reflect isolated renal vulnerability, but rather a broader syndrome of multiorgan cross-talk driven by ventilatory pressures.

We acknowledge that higher P_mean_ may partly reflect greater illness severity and more severe lung disease, which are themselves associated with AKI. However, the association remained consistent after adjustment for multiple severity scores and cardiopulmonary/hemodynamic proxies and was robust across complementary analyses in two independent databases. Thus, while causality cannot be inferred, P_mean_ may capture a clinically relevant component of ventilatory load beyond measured severity alone. We also acknowledge that ‘optimal PEEP’ strategies have been extensively studied and peak pressure limitation is long-standing practice. The novelty of our work is to position P_mean_ as an integrative exposure reflecting the combined influence of PEEP, inspiratory pressure, and inspiratory timing (I:E), and to provide inflection points that may inform stratification in future prospective studies. Future trials could compare different combinations of these settings that achieve similar gas exchange but different P_mean_ profiles to test whether reducing sustained intrathoracic pressure burden improves renal outcomes.

Despite providing robust multicenter evidence of an association between higher P_mean_ and AKI, several limitations are inherent to the retrospective design and data sources. First, as an observational analysis, it can establish association but not causality. In addition, ICU practices may differ across centers and databases, including ventilation strategies, fluid management, and AKI surveillance, and such heterogeneity is unavoidable and not fully quantifiable. Second, several clinically relevant variables were incompletely captured, including static compliance and formal ARDS criteria, which required the use of surrogate measures; ventilator mode information was not extractable in eICU-CRD and was available only for a subset in MIMIC-IV, and CVP and echocardiography variables were not systematically available and could not be incorporated into the main analyses. Third, although we performed comprehensive adjustment, residual confounding may persist, including unmeasured factors such as details of major surgical procedures, incomplete characterization of chronic kidney disease severity, and indication bias related to lung disease severity. Moreover, fluid responsiveness and venous congestion could not be directly measured in the full cohort, and ventilatory parameters are physiologically interrelated, limiting complete separation of their individual contributions. Fourth, sample attrition in eICU-CRD and the long study time span, together with different calendar periods covered by the two databases, may introduce secular bias related to changes in ICU care over time. Finally, the assessment of nephrotoxic exposures was limited by their low recorded prevalence. Notwithstanding these limitations, the consistent, dose-dependent relationship observed across two independent databases and the robustness of findings to multiple analytical approaches strongly support the validity of our conclusions.

## 5. Conclusion

This multicenter study demonstrates that elevated P_mean_ is independently associated with AKI in critically ill patients receiving mechanical ventilation. Using two independent ICU databases, we consistently observed a dose–response relationship between higher P_mean_ and increased AKI risk. After comprehensive adjustment for confounding factors, a P_mean_>8.7 cmH_2_O was independently associated with worse clinical outcomes, including greater AKI severity, higher utilization of renal replacement therapy, increased mortality, and prolonged ICU length of stay. While elevated P_mean_ may partly reflect greater illness severity and causality cannot be inferred from retrospective data, our findings provide clinically operational information that may inform eligibility criteria and stratification in future prospective and randomized studies. Rather than re-testing single-parameter strategies alone, future trials could evaluate P_mean_ -guided approaches—including combinations of PEEP, inspiratory pressure targets, and inspiratory timing (I:E ratio)—to determine whether minimizing sustained intrathoracic pressure burden can improve renal outcomes without compromising gas exchange.

## Supplementary Material

Supplemental Material

## Data Availability

The data used in this study are available from the MIMIC-IV database and the eICU Collaborative Research Database *via* PhysioNet (https://physionet.org/), subject to completion of the required data use agreement and training. All raw data can be obtained through the corresponding author, Keliang Xie and first author Ying Gao, upon reasonable request.

## Abbreviations

P_mean_: Mean Airway Pressure
AKI: Acute Kidney Injury
MIMIC-IV: Medical Information Mart for Intensive Care IV
RCS: Restricted Cubic Spline
PSM: Propensity Score Matching
ICU: Intensive Care Unit
MAP: Mean Arterial Pressure
CVP: Central Venous Pressure
SOFA: Sequential Organ Failure Assessment (SOFA)
APS III: Acute Physiology Score III
APACHE II: Acute Physiology and Chronic Health Evaluation II
PEEP: Positive End-Expiratory Pressure
OR: Odds Ratio
CRRT: Continuous Renal Replacement Therapy
MV: Mechanical Ventilation
LOS: Length Of Stay
CI: Confident Interval
VILI: ventilator-induced lung injury
