# Supplementary figures and images for: Mean airway pressure as an independent risk factor for developing acute kidney injury in mechanically ventilated critically ill patients: a multi-center retrospective analysis
Source: Ren Fail. 2026 May 24;48(1):2675108. doi: 10.1080/0886022X.2026.2675108 (PMC13202667; doi:10.1080/0886022X.2026.2675108)

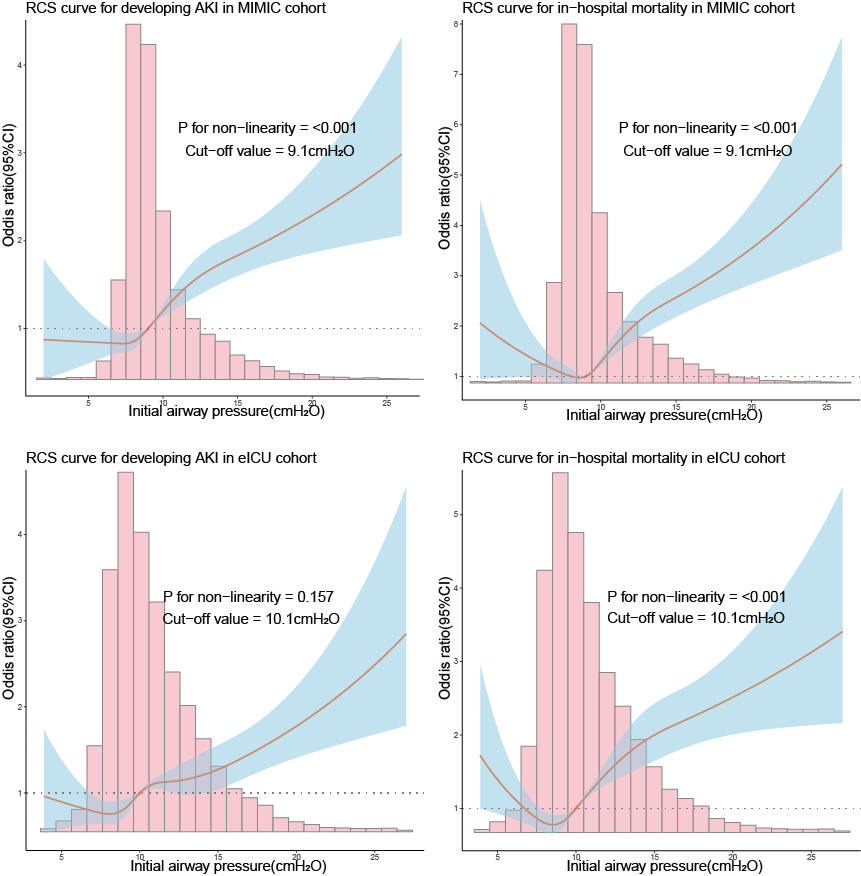

Supplement: Supplemental Material [file IRNF_A_2675108_SM9179.tif]
